# SARS-CoV-2 infection serology: a useful tool to overcome lockdown?

**DOI:** 10.1038/s41420-020-0275-2

**Published:** 2020-05-26

**Authors:** Marzia Nuccetelli, Massimo Pieri, Sandro Grelli, Marco Ciotti, Roberto Miano, Massimo Andreoni, Sergio Bernardini

**Affiliations:** 1 Tor Vergata University Hospital, Rome, Italy; 2 Department of Experimental Medicine, University of Tor Vergata, Rome, Italy; 3 Department of System Medicine, University of Tor Vergata, Rome, Italy; 4 IFCC Emerging Technologies Division, Milan, Italy; 5 Department of Surgical Sciences, University of Tor Vergata, Rome, Italy

**Keywords:** Viral infection, Immunochemistry

## Abstract

The outbreak of 2019 novel coronavirus disease (Covid-19) caused by SARS-CoV-2 has spread rapidly, inducing a progressive growth in infected patients number. Social isolation (lockdown) has been assessed to prevent and control virus diffusion, leading to a worldwide financial and political crisis. Currently, SARS-CoV-2 RNA detection in nasopharyngeal swab takes place by real-time PCR (RT-qPCR). However, molecular tests can give some false-negative results. In this context, serological assays can be useful to detect IgG/IgM antibodies, to assess the degree of immunization, to trace the contacts, and to support the decision to re-admit people at work. A lot of serological diagnostic kits have been proposed on the market but validation studies have not been published for many of them. The aim of our work was to compare and to evaluate different assays analytical performances (two different immunochromatographic cards, an immunofluorescence chromatographic card, and a chemiluminescence-automated immunoassay) on 43 positive samples with RT-qPCR-confirmed SARS-CoV-2 infection and 40 negative control subjects. Our data display excellent IgG/IgM specificities for all the immunocromatographic card tests (100% IgG and 100% IgM) and for the chemiluminescence-automated assay (100% IgG and 94% IgM); IgG/IgM sensitivities are moderately lower for all methods, probably due to the assay viral antigen’s nature and/or to the detection time of nasopharyngeal swab RT-qPCR, with respect to symptoms onset. Given that sensitivities (around 94% and 84% for IgG and IgM, respectively) implicate false-negative cases and given the lack of effective vaccines or treatments, the only currently available procedure to reduce SARS-CoV-2 transmission is to identify and isolate persons who are contagious. For this reason, we would like to submit a flowchart in which serological tests, integrated with nasopharyngeal swab RT-qPCR, are included to help social and work activities implementation after the pandemic acute phase and to overcome lockdown.

## Introduction

In early December 2019, a novel human coronavirus was identified as the agent responsible for the first pneumonia cases of unknown origin in Wuhan, Capital of Hubei Province, China^1^. The virus was classified as a wrapped RNA Betacoronavirus2, which was readily called SARS-CoV-2 (severe acute respiratory syndrome coronavirus 2)^2,3^.

The SARS-CoV-2 infection causes Coronavirus Disease 2019 (Covid-19), documented both in hospitals and in home structures^1^. The World Health Organization (WHO), on January 12, 2020, declared Covid-19 as a public health emergency of international concern. On March 11, WHO assessed that Covid-19 can be characterized as a pandemic. The SARS-CoV-2 infection has been extremely contagious, with over 2,314,621 infected people and 157,847 death cases confirmed in laboratories, since April 20, 2020^4^. It has been rapidly spreading all over the world with minor local differences, inducing a progressive growth in the number of patients who need access to emergency departments and an increasing demand in diagnosis of SARS-CoV-2 infection^5^.

The Covid-19 pandemic is more than a public health emergency; it is a financial and political crisis afflicting every nation in the world. To date, millions of people stay at home worldwide, to minimize transmission of SARS-CoV-2, except healthcare workers and workers employed in essential services such as food production, transport and delivery, police, firefighters, and others^2,6,7^.

In the meantime that social isolation (lockdown) prevents and controls the pandemic diffusion to mitigate SARS-CoV-2 spreading, States economies get in serious troubles and many governments are looking to find a good balance between prevention of SARS-CoV-2 disease and a “soften up” lockdown, to restart industrial production and limit Gross Domestic Product loss. In the absence of a vaccine, Authorities have to find a way to reach the best compromise between Covid-19 prevention and lockdown economic and social impact^8^. Indeed, it is well known that isolation in the elderly may increase the risk of cardiovascular, autoimmune, and neurocognitive diseases, together with mental health problems; not only these, but many other important social aspects of people’s daily life have to be considered: education, stress and family conflicts, and job lost^9–11^. Schools have been suspended nationwide in 188 countries, according to the United Nations Educational, Scientific and Cultural Organization and over 90% enrolled learners (1.5 billion young people) are now out of education. It should be considered that children’s daily routines are important, in particular for those with special needs, e.g., children with autism. Children and adolescents living in isolation, in abusive homes, are in danger during this time of economic uncertainty and stress^12^. Finally, distance-learning educational activities are not available to everyone and are a source of inequity worldwide^13^.

Moreover, how to manage surgical patients during Covid-19 pandemic is still a matter of debate, with a tremendous impact on the surgery units’ organization and productivity^14^. To date, all patients must be judged as potentially positive, in consideration of the high estimated number of asymptomatic subjects and of the maximum contagiousness that occur 2–3 days immediately preceding the onset of symptoms. Recently, it has been shown that surgery performed on patients unaware of being infected and in the incubation phase determines a 20% increasing risk in postoperative mortality and 50% in post-surgery intensive care unit hospitalization, possibly because surgery led to an inflammatory process acceleration causing Covid-19 progression^15^.

Currently, SARS-CoV-2 RNA detection in the nasopharyngeal swab or bronchoalveolar lavage (BAL), together with some hematological parameters and chest computed tomography (CT), are the primary tools for confirmation of Covid-19 clinical suspicion^8^. Detection of viral nucleic acid takes place by real-time quantitative PCR (RT-qPCR) method inside authorized laboratories, with biological safety class 2. However, RT-qPCR kits can give some false-negative results, depending on swab sampling and extraction method, and on the possibility that virus, even if present in the individuals, is not detectable in nose–pharynx mucous membrane^16^. Recently, some studies described a discrepancy between the diagnostic power of RT-qPCR and CT, the latter being more sensitive^8^. Incidence of false negatives at molecular tests sometimes force repetition of the same, up to three times in clinically suspected Covid-19 patients and/or with a Covid-19 CT scan pattern^8^. New, more user-friendly molecular tests are on the horizon for “out of the Lab” SARS-CoV-2 RNA screening, utilizing Heating Unextracted Diagnostic Samples Obliterate Nuclease and cards to run Clusters of Regularly Interspaced Short Palindromic Repeats (CRISPRs) methods, as DNA Endonuclease Targeted CRISPRs Trans Reporter^17^. In this context, a great debate is ongoing about the role of serological assays able to detect IgG, IgA, or IgM anti-SARS-CoV-2 in serum, plasma, or capillary blood, to have a clear picture of the outbreak size in each country, to assess the degree of immunization and to support the decision to re-admit people at work^18^. Several serological assays have been developed since the beginning of Covid-19 pandemic, including enzyme-linked immunosorbent assays (ELISA), rapid antibody immunochromatographic tests, point-of-care test (POCT)-fluorescence assays, and chemiluminescence immunoassays (CLIAs)^18^. More than 120 different diagnostic kit brands have been proposed on the market, many are CE (European Community) approved; to date, very few are Food and Drug Administration (FDA) approved^19,20^. Serological tests to detect antibodies against viral antigens are not yet widely used during this pandemic, but rather in a “leopard spot” manner in publics and private laboratories; however, they could be as useful as they were during SARS epidemic of 2002^21^ and some studies demonstrate the anti-SARS-CoV-2 IgG/IgM presence in clinically confirmed cases with negative RT-qPCR results^22^. Serological tests are cheaper than molecular tests, require a shorter analytical time, and productivity can be much greater than molecular tests in case of automated instruments in medium-large hospital laboratories, employing CLIA and ELISA.

Serological tests for specific SARS-CoV-2 antibodies detection in patient’s blood are currently important to: (a) trace contacts; (b) activate serological surveillance at the local, regional, and national level; and (c) identify those who have already had contact with virus^23^. Assuming there is protective immunity, serological information can be used to decide return at work of infected workers, especially people who work in environments in which they can potentially be exposed to SARS-CoV-2 (e.g., health professionals). Unfortunately, majority of in vitro diagnostic (IVD) companies do not report nature of the antigen/s utilized in the assays and then it is difficult to understand whether antibodies detected with different kits and methodologies have a neutralizing effect on the virus, possibly through binding with the viral spike protein S subunits receptor-binding domain (RBD)^24,25^. In addition, serological tests can be used retrospectively in post-mortem diagnosis and, finally, they can be eventually used together with molecular tests for improving their diagnostic accuracy. Furthermore, in the near future, serological tests could play a role in the efficacy evaluation of any identified vaccines^26^. Although IgM antibodies generation may occur as rapidly as the viral genetic material in the respiratory tract, generally the timing of immunoglobulin production (from 4 days after the onset of symptoms, to 10–14 days) limits its applicability in the acute phase diagnosis^27,28^. Nevertheless, it should be pointed out that molecular tests represent an “instantaneous” picture of possible virus presence, whereas serological tests display virus presence during a wider phase of the infectious process, whether or not it reaches a clinical relevance. Moreover, in particularly serious cases, the use of serological tests in titration of convalescent patients hyperimmune plasma cannot be excluded (once the antibodies ability to neutralize the virus has been established), after considering all possible side effects of this treatment^29^. About that, FDA is coordinating a national effort to develop blood-based antibody-rich Covid-19 therapies. Serological tests, through the study of humoral response profile and clinical observations, may also contribute to definition of IgG serum concentration suitable for a subject to be immune, as well as the persistence time of any immunization.

Based on all the above considerations, the aim of this study was to compare and to evaluate different serological tests diagnostic accuracy (two different immunochromatographic card tests, an immunofluorescence chromatographic card test, and a chemiluminescence-automated immunoassay test), able to detect anti-SARS-CoV-2 IgG and IgM in healthy controls and in Covid-19 patients, monitored at “Tor Vergata” University Covid-Hospital of Rome.

## Results

**Fig. 1:**
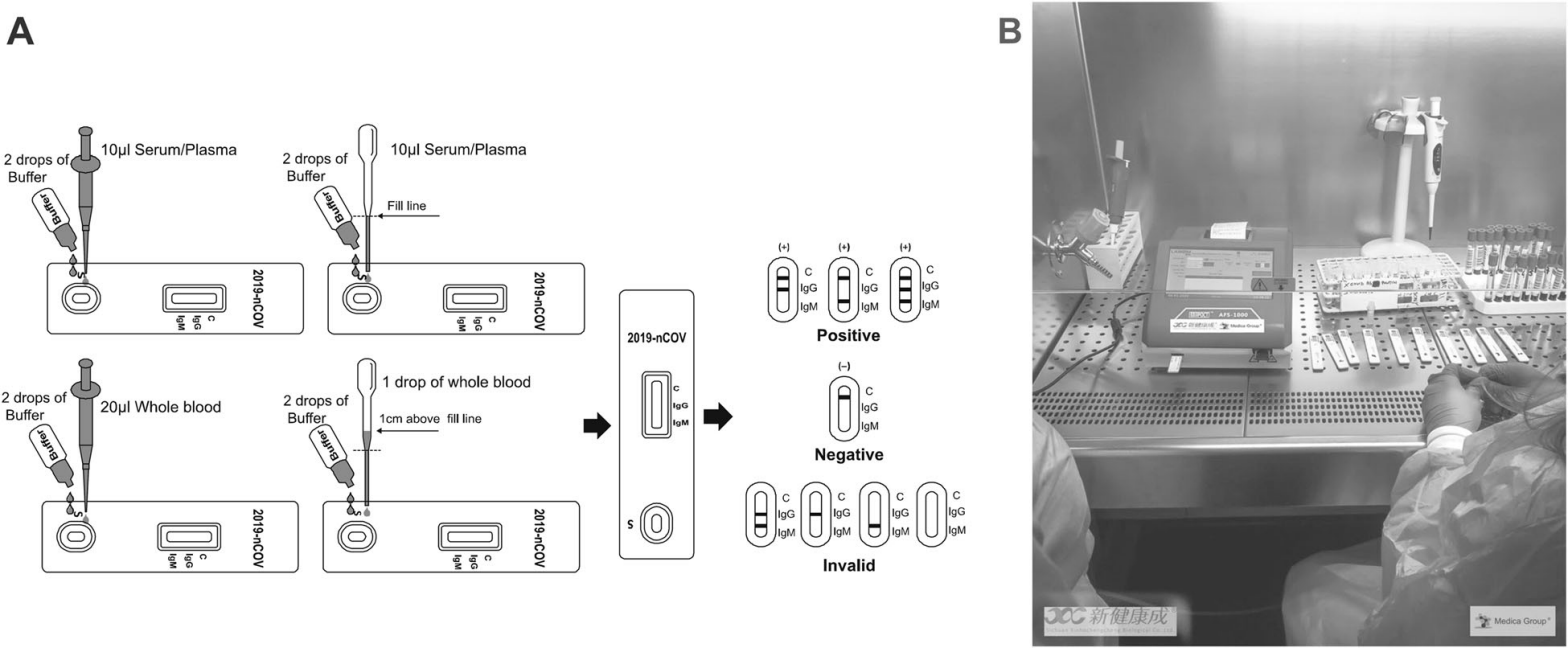
Schematic of SARS-CoV-2 Immunochromatographic and immunofluorescence Card Test workflow and assay readout. **a** Three detection lines are on the test cassette: a control (c) line appears when serum sample flows through the card; SARS-CoV-2 antibodies presence will be indicated by a colored line in the IgG and/or IgM test line regions. If C line does not appear, the test is invalid and should be repeated. **b** Samples, mixed with a buffer solution containing fluorescently labeled SARS-CoV-2 recombinant proteins, form antigen–antibody conjugates. The conjugates are added to the sample well in the test card and captured by nitrocellulose-coated goat-anti-human IgM or IgG antibodies. The resulting immunocomplexes are detected by a fluorescence detector. Positive cut-off values are as follows: anti-SARS-CoV-2 IgG positive>1,2T/C; anti-SARS-CoV-2 IgM positive > 1,3T/C.

From March 16 to 23 April, 2020, in our hospital were performed 9414 nasopharyngeal swabs (mean age 55.4 ± 19.0 years), with 1085 RT-qPCR SARS-CoV-2-positive results (mean age 62.1 ± 18.7 years). At first, Card 1 was the only available method for serological screening of nurses, physicians, and other healthcare workers. The test was performed on about 1200 people, and to evaluate Card 1 serological specificity and sensitivity, we collected blood samples related to people analyzed by RT-qPCR, at least after 4 days from the execution of the nasopharyngeal swab. We selected 307 samples as follows: 82 with RT-qPCR nasopharyngeal-positive swabs (mean age 63.4 ± 15.7 years) and 225 with RT-qPCR nasopharyngeal-negative swabs (mean age 54.5 ± 19.5 years). On this cohort, specificity was 100% but sensitivity was lower: 53.6% and 76.9% for IgM and IgG, respectively. Although commercial manufacturers claim their tests have high sensitivity and specificity, many of them have not published “on field” validation studies yet. Later on, with other tests becoming available, we decided to compare the anti-SARS-CoV-2 IgG and IgM detection sensitivity and specificity among different methods and kits: two immunochromatographic card tests (named Card 1 and Card 2; Fig. 1a), one immunofluorescence chromatographic card test (Card 3; Fig. 1b), and one chemiluminescence-automated immunoassay (CLIA). At this purpose, we collected 43 positive samples from Covid-19 patients with RT-qPCR-confirmed SARS-CoV-2 infection (mean age 63.1 ± 13.0 years) and 40 control subjects, negative to nasopharyngeal swabs RT-qPCR (mean age 49.9 ± 12.8 years).

Specificity and sensitivity were calculated using receiver operating characteristic (ROC) curves, for semi-quantitative tests, and with formulas reported under “Materials and Methods,” for qualitative cards. Results with the analytical parameters for each test (area under curve (AUC), sensitivity, and specificity) are shown in Table 1. They have been correlated to the manufacturer’s cut-off and recalculated on a best fit cut-off that emerged from our data analysis.

**Table1:** Area under curve (AUC), sensitivity, and specificity of SARS-CoV-2 IgG–IgM serological tests.

Positive RT-qPCR sample: 43 Negative RT-qPCR sample: 40	CARD 1		CARD 2		CARD 3		CLIA	
	IgM	IgG	IgM	IgG	IgM	IgG	IgM	IgG
Sensitivity (%)	61.4	84.3	87.8	89.6	84	93	84	95
Specificity (%)	100	100	100	100	100	100	94	100
Kit cut-off	Qualitative test				>1.3 T/C	>1.2 T/C	>1 COI	>10 COI
Area under the ROC curve (AUC); 95% confidence interval					0.921; 0.830 to 0.969	0.981; 0.918 to 0.999	0.943; 0.863 to 0.983	0.997; 0.945 to 1.000
Sensitivity (%)					84	93	91	95
Specificity (%)					100	100	94	100
Laboratory cut-off					>1.28 T/C	> 1.2 T/C	> 0.54 COI	> 10.26 COI

**Fig. 2:**
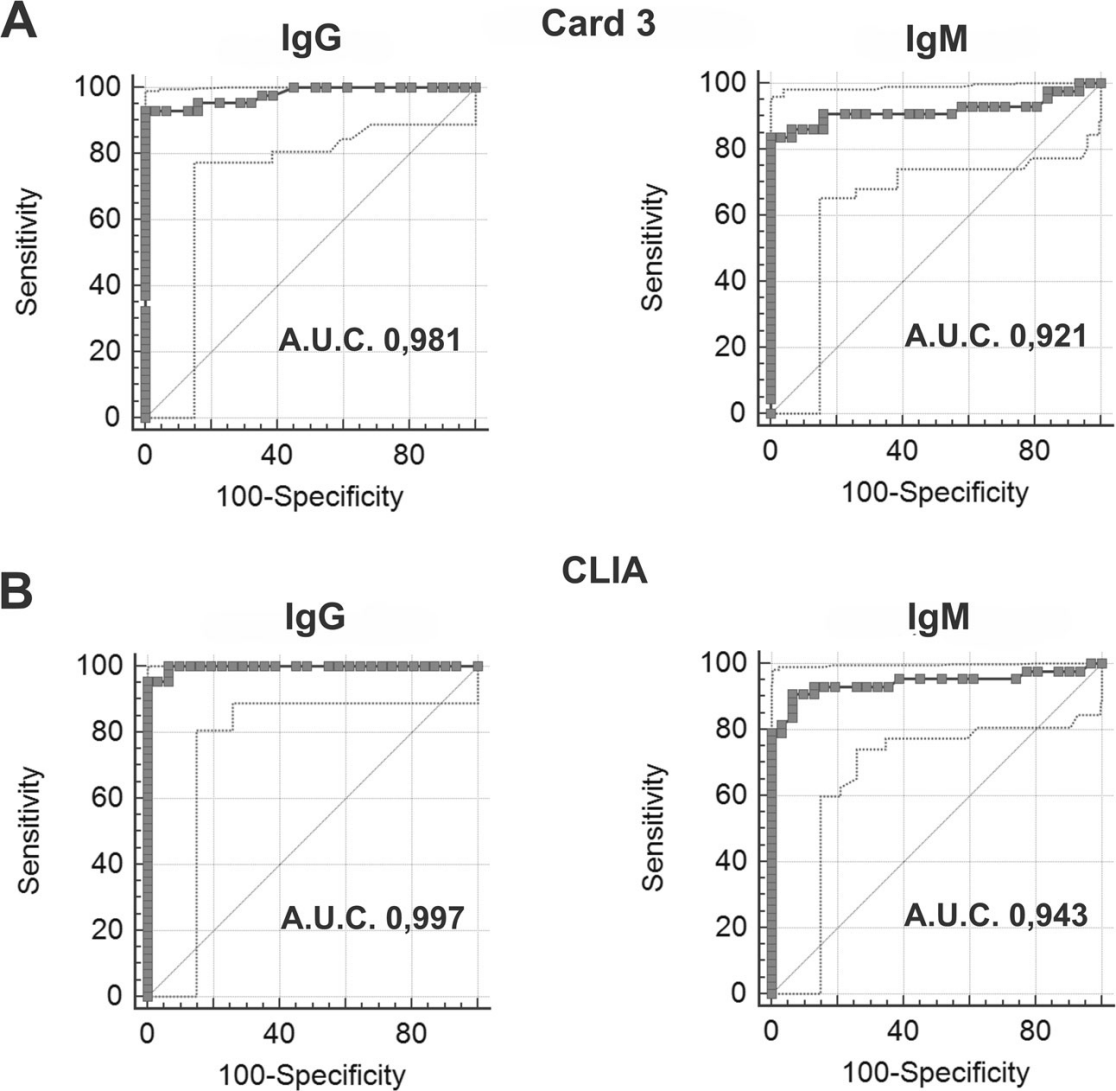
Anti-SARS-CoV-2 serological tests ROC curves. Card 3 IgG and IgM results are shown in **a** (AUC 0.981 and 0.921, respectively); CLIA IgG and IgM results are shown in **b** (AUC 0.997 and 0.943, respectively).

The ROC curves have optimal AUC values (close to 1) for IgG detection in Card 3 and CLIA (0.981 and 0.997, respectively); AUC values for IgM detection are moderately lower (0.921 and 0.943, respectively; Fig. 2).

Moreover, our data displays an excellent specificity for all the immunocromatographic tests (100%) with a slight decrease in the IgM specificity of CLIA (94%); on the other hand, IgG sensitivities are about 90% for all tests, with better performances for Card 3 and CLIA (93% and 95%, respectively); IgM sensitivities are lower (around 84%), with a best performance of Card 2 (87.8%) and a low value for Card 1 (61.4%). Interestingly, with our recalculated best fit cut-off (0.54 cutoff index (COI) instead of 1 COI), CLIA IgM sensitivity increased from 84% to 91%.

## Discussion

Due to the rapid spreading of Covid-19 pandemic, many molecular and serological detection tools have been rapidly developed^8,18,26,30–33^. Laboratory confirmation of SARS-CoV-2 infection was based on RT-qPCR-positive results^34^. However, molecular tests carried out through swabs can also be negative in people who harbor the virus, because swab collection and time of sampling are critical; in severe cases, the most suitable samples, showing a higher RNA-positive rate, seem to be BAL or deep sputum^35^ and WHO recommends to repeat a negative test under a strong clinical suspicion.

Moreover, the great sanitary and social pressure increased the request in the number of tests carried out in laboratories, leading to a worldwide shortage of kits and reagents. Therefore, today IVD companies are getting in trouble, in delivering an adequate number of tests, to cover all laboratories needs. For this reason, WHO and Centers for Disease Control and Prevention recommend a prioritization of testing policy^36^. Of course, the sustainability grade of Covid-19 screening in different countries may determine serious inequities.

The most used viral proteins as antigens in the available serological assays are as follows: nucleocapsid protein (N), transmembrane spike protein (S), or protein S subunits RBD^37^. Whatever the method used, nature of the antigen is important, considering that detection of antibodies directed against spike protein or its subunits are more likely to have a neutralizing activity and would better describe the immunization state. According to these observations, our samples higher sensitivities were obtained with the assays based on the presence of both, N and S proteins. Therefore, this information should be reported on kit datasheets, but several times it is missing.

Based on our good analytical performance results for all tested methods, it should be pointed out that chemiluminescence assays are fully automated and can be run in total automation laboratories, also limiting technologists exposition to blood samples. About laboratory safety of technologists engaged in routine hematology/biochemistry, we are confident that these procedures are not deemed to be aerosol generating. Moreover, viraemia does not appear to be a significant feature of mild-to-moderate Covid-19 and where viral RNA has been detected, this has been at a low level. Nevertheless, viraemia may be a feature in more severe Covid-19, although current data are conflicting. About that, however, it is strongly recommended to wear face masks, gloves, disposable coats, and protective glasses (mandatory safety set in Italian clinical laboratories). Regarding immunochromatographic cards, they are fully manual and the reading is subjective, whereas immunofluorescence cromatographic cards set up is manual but the reading is accomplished through a fluorescence detector. Traceability is complete in CLIA and in fluorescence assays; furthermore, both of them are able to send results directly to Laboratory Information System. We strongly suggest to revise the cut-off values reported on datasheets by companies, because sometimes they are calculated in a small number of subjects belonging to a specific ethnicity or region. In addition, given the large number of certified companies able to produce and distribute serological kits, each of them should be first tested and validated by accredited laboratories.

In each context, relying on the instruments, on the available economic investments and on the wideness of screenings, all different methods described can be utilized considering the limits of each one. For example, screening sustainability should take into account the cost/benefit ratio, assuming the cost of one immunochromatographic card (IgG + IgM) as 1.0, the cost of an immunofluorescence card as 1.5 (IgG + IgM) and the cost of CLIA (IgG + IgM) as 2.0. Nevertheless, different strategies can be chosen depending on the target: support to diagnosis with IgM, or serological screening and epidemiology studies with only IgG. Finally, considering the advantage that immunochromatographic cards are ready-to-use and time-saving^38^, in some contexts may be easier to analyze capillary blood rather than collect, centrifuge, transport, and store blood samples (airports, prisons, religious communities, sports associations, and centers for the elderly). In our study, we used serum also in the immunocromatographic cards, so we cannot completely exclude different performances with capillary blood.

At present, most countries are in some form of lockdown, with journeys severely restricted and reduced to essential trips only. These security measures reduce disease transmission by reducing the number of susceptible persons in the population or by reducing the basic reproductive number (R0) that is modulated by such factors as duration of viral shedding, infectiousness of the organism, individuals at stage I (stealth carriers), and contacts matrix between infected and susceptible persons^39^. Moreover, given that serological assay sensitivities (in our study around 94% and 84% for IgG and IgM, respectively) implicate a number of false-negative cases and given the lack of effective vaccines or treatments, the only currently available procedure to reduce SARS-CoV-2 transmission is to identify and to isolate persons who are contagious.

**Fig. 3:**
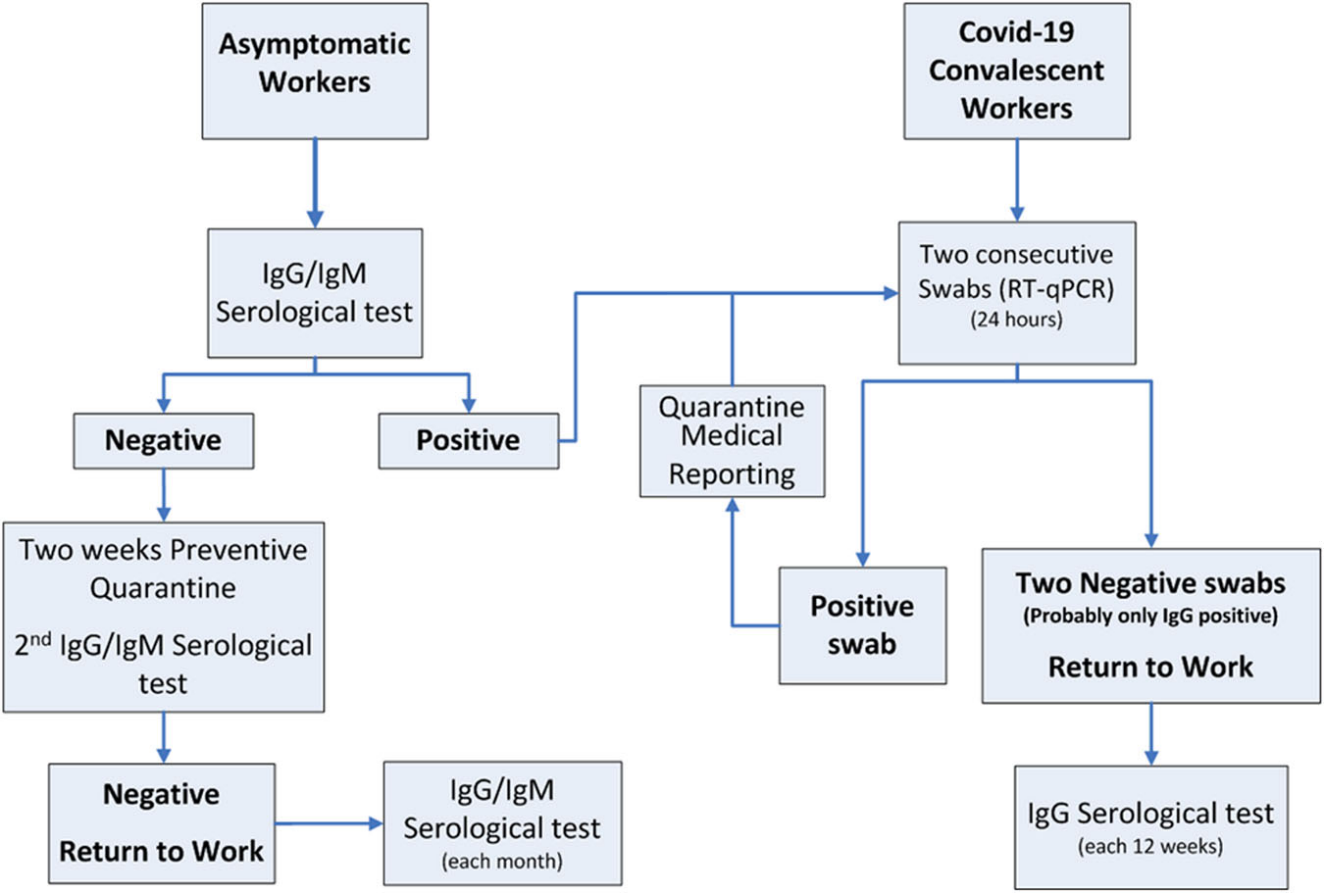
Flowchart proposal to overcome lockdown. Path 1 describes serological IgG/IgM assays on asymptomatic workers: in case of negative results, after a 2-week preventive quarantine, a second negative IgG/IgM serological test is required to return to work; in case of positive results, two consecutive nasopharyngeal swabs RT-qPCR are mandatory. Path 2 describes serological IgG assays on Covid-19 convalescent workers or IgG/IgM-positive asymptomatic workers, after two consecutive negative nasopharyngeal swabs RT-qPCR, to detect natural immunization span.

At this purpose, we would like to submit a proposal for a flowchart in which serological tests are included to help social and work activities implementation after the pandemic acute phase (Fig. 3). The flowchart shows two different paths as follows: in the first, there are subjects who have always been asymptomatic; in the second, there are patients who have overcome the disease. Serology is integrated with nasopharyngeal RT-qPCR swab when necessary, to exclude topical virus presence. It is true that there is no 100% guarantee of virus-free among the groups tested with serological and molecular assays, but in the flowchart we propose a 2-week preventive quarantine and a second serological IgG/IgM assay before re-admitting people at work. Taking into account that no test method is perfect and there are still a lot of unknown elements for this virus, social distancing should be kept for a longer time, especially before a valid vaccine or therapy are established. In fact, it will be still highly suggested that people finally labeled as “return to work,” should continue to use personal protections such as face mask, frequent hand-washing, and keeping necessary physical distances with each other. The median day of serum conversion for both IgG and IgM has been calculated 13 days after symptom onset^27,40^, so the preventive security measures, together with the double IgG and IgM serological testing (one at the beginning of the quarantine and one after 2 weeks), can be helpful for recovery of activities in less time and in safety. Obviously, the span of natural immunization is still unknown and will be determined in the future, through clinical and serological follow-up; for this reason, we prudently suggest a time of 3 months for testing IgG-positive subjects, considering a SARS-CoV-2 epidemic duration of at least 6 months (reported during 2002 SARS epidemic)^41^.

Finally, prevalence is currently determined on the number of subjects with positive RT-qPCR nasopharyngeal swabs, then the real prevalence is probably much higher. If we compare the most affected Northern Italy region Covid-19 prevalence (Lombardia: 0.67%), to a Center Italy region prevalence (Lazio: 0.09%), the positive predictive value of serological tests would be extremely low in the second case. Serological screening should therefore be performed first on an epidemiologically significant population sample, to define the usefulness of a more massive screening^42^. In Italy, such population serological sample screening (150,000 subjects) will be carried on by May 2020, to establish Covid-19 prevalence in each different region and to support the management of the so-called “phase two.”

## Conclusions

The flowchart proposed in this work emphasize the importance of serological tests and manage resources for resuming activities and overcoming lockdown.

In particular, for wide screenings through blood sampling, including surgical patients, we recommend to adopt CLIA methods in large hospital laboratories, working in total automation; immunochromatographic cards could be utilized during sampling in specific contexts outside the hospitals such as follows: high prevalence areas; airports; police and military forces stations; prisons; immigrants, homeless, religious and other communities. Nevertheless, from a public health perspective, testing for IgG anti-SARS-CoV-2 presence could determine who has been exposed, to better define the possibility of asymptomatic infections and to give us a more reliable case counts and mortality estimates, and a useful tool to control the restarting phase.

Lastly, in a very less time, all countries will be challenged by “phase two,” but until the neutralizing effect of detected antibodies with all different methods and antibody serum level needed to be fully protected towards a re-infection, will be definitively reported, we could not consider serological positivity as a “license” to quit social distance rules and protective devices.

## Materials and methods

### Patients and serum specimens

Serum samples were collected from RT-qPCR-diagnosed SARS-CoV-2-positive (*n* = 43) and negative (*n* = 40) patients from “Tor Vergata” University Covid-Hospital of Rome, in accordance with local ethical approvals (R.S.44.20). Informed consent was obtained from all subjects enrolled in the study. Sera were separated by centrifugation at 2500 × *g* for 10 min, within 1 h from collection. All serum samples were collected at least 4 days after nasopharyngeal swab. The study was in accordance with the Helsinki Declaration, as revised in 2013.

### Real-time PCR (RT-qPCR)

Nasopharyngeal swabs were tested for SARS-CoV-2 infection with Seegene AllplexTM2019-nCoV Assay (Seegene, Seoul, South Korea), according to the manufacturer’s protocols.

Automated RNA extraction and PCR setup were carried out using Seegene NIMBUS, a liquid handling workstation. RT-qPCR was run on a CFX96TMDx platform (Bio-Rad Laboratories, Inc., CA, USA) and subsequently interpreted by Seegene’s Viewer Software. The Seegene AllplexTM2019-nCoV Assay identifies the virus by multiplex real-time PCR targeting three viral genes (*E*, *RdRP* and *N*), thus complying with international validated testing protocols.

### Immunochromatographic card test 1: Card 1

Lateral flow chromatographic immunoassay for qualitative detection of IgG and IgM antibodies to 2019-nCoV in human whole blood, serum, or plasma specimens (2019-nCoV IgG/IgM Rapid Test Cassette, Hangzhou AllTest Biotech Co, Hangzhou, China; distributed in Italy by Alifax Srl, Padova, IT). During testing, sample reacts with 2019-nCoV antigen-coated particles (recombinant N-Protein, as declared by manufacturer) in the test cassette and a colored line will appear in IgG or IgM test line region as a result, and in the control region (C) as an internal procedural control. Results are read after 10 min incubation; it should not exceed 20 min. This test is CE approved.

### Immunochromatographic card test 2: Card 2

Lateral flow immunoassay for qualitative detection of IgM and IgG antibodies to SARS-CoV-2 in serum, plasma (EDTA, citrate) or whole blood specimens (Cellex qSARS IgG/IgM Rapid Test, Cellex, Inc., NC, USA; distributed in Italy by Alifax Srl, Padova, IT). The test cassette consists of a colored conjugate pad containing SARS-CoV-2 recombinant antigens (N-Protein and Spike Protein (S), as declared by manufacturer) conjugated with colloidal gold (SARS-CoV-2 conjugates) and rabbit IgG-gold conjugates; a nitrocellulose membrane strip containing an IgG line (G Line) coated with anti-human IgG, an IgM line (M Line) coated with anti-human IgM, and the control line (C Line) coated with goat-anti-rabbit IgG. Results are read after 15 min incubation; it should not exceed 20 min. This test is FDA and CE approved.

### Immunofluorescence chromatographic card test: Card 3

POCT-fluorescence Coronavirus IgG/IgM antibodies detection kit (Sichuan Xincheng Biological Co., China; distributed in Italy by Medica Group, Rome, IT). Immunofluorescence chromatography method for semi-quantitative determination of SARS-CoV-2 IgG/IgM antibodies in human whole blood (capillary blood), serum, and plasma. Sample is uniformly mixed with a buffer solution and the mixture reacted with fluorescently labeled SARS-CoV-2 recombinant proteins (N-Protein and Protein S RBD, as declared by the manufacturer) to form an antigen–antibody conjugate. The conjugate is added dropwise to the sample well in the test card and captured by nitrocellulose-coated goat-anti-human IgM or IgG antibodies. The resulting immunocomplexes are detected by a fluorescence detector (AFS-1000 Immunofluorescent Analyzer, Guangzhou Labsim Biotech Co., Guangzhou, China; distributed in Italy by Medica Group, Rome, IT), to achieve a semi-quantitative determination of anti-SARS-CoV-2 antibodies concentration and allowing sample traceability. Results are read after 8 min incubation; do not exceed 10 min. Positive cut-off values, according to the manufacturer’s instructions are as follows: anti-SARS-CoV-2 IgG positive > 1.2 T/C; anti-SARS-CoV-2 IgM positive > 1,3 T/C. This test is CE approved.

### Chemiluminescence immunoassay

The CL-series SARS-CoV-2 IgG and IgM assays are a two-step chemiluminescent immunoassays for detection of IgG and IgM SARS-CoV-2 antibodies in human serum or plasma, performed on the fully automated Mindray CL 1200i analytical system (Shenzhen Mindray Bio-Medical Electronics Co., Shenzen, China; distributed in Italy by Medycal System, Genova, IT). Samples react with paramagnetic microparticles coated with SARS-CoV-2 specific antigens (recombinant N-Protein and Spike (S) Protein, as declared by the manufacturer). Alkaline phosphatase-labeled anti-human IgG or IgM monoclonal antibodies are added to the reaction to form sandwich with microparticles captured anti-SARS-CoV-2 antibodies. Finally, a substrate solution is added, resulting a chemiluminescent reaction measured as relative light units by a photomultiplier built into the system. First results are generated after 25 min (throughput 180 tests/h). Cut-off values are: IgG positive > 10 COI and IgM positive >1 COI, according to the manufacturer’s instructions. Kits were provided as Research Use Only (RUO), for oversea validation.

### Statistical analysis

Qualitative rapid test kits specificity and sensitivity were calculated according to the following formulas:

Specificity (%) = 100 × [True negative/(True Negative + False Positive)].

Sensitivity (%) = 100 × [True Positive/(True Positive + False Negative)]

Specificity and sensitivity for Card 3 and CLIA were calculated by ROC curves. All data were analyzed using Med Calc Ver.18.2.18 (MedCalc Software Ltd, Ostend, Belgium). The investigator was blinded to the group allocation during the experiment.
